# Stress and Turnover Intentions Within Healthcare Teams: The Mediating Role of Psychological Safety, and the Moderating Effect of COVID-19 Worry and Supervisor Support

**DOI:** 10.3389/fpsyg.2021.758438

**Published:** 2022-01-31

**Authors:** Melany Hebles, Francisco Trincado-Munoz, Karina Ortega

**Affiliations:** ^1^Administration Department, Universidad Catolica de la Santisima Concepcion, Concepcion, Chile; ^2^Business School for the Creative Industries, University for the Creative Arts, Epsom, United Kingdom

**Keywords:** psychological safety, turnover intentions, COVID-19, cognitive stress, supervisor support

## Abstract

Employees at healthcare organizations are experiencing more stress than ever given the current COVID-19 pandemic. Different types of stress are affecting diverse organizational outcomes, including the employees’ voluntary turnover. This is the case of cognitive stress, a type of stress that affects how individuals process information, which can influence employees’ turnover intentions. In this study, we look at the mechanisms that can reduce the adverse effects of cognitive stress on turnover intentions, particularly the role of employees’ perceived psychological safety (i.e., how safe they perceive the interactions with their colleagues are). We hypothesize that psychological safety mediates the relationship between cognitive stress and turnover intentions, and COVID-19 worry and supervisor support moderate the relationship between cognitive stress and psychological safety. To test our hypothesis, we invited two public health care organizations in Chile to join this study. In total, we obtained a sample of 146 employees in 21 different teams. Using a multilevel model, we found that psychological safety prevents the harmful effects of cognitive stress on employees’ turnover intentions. In addition, while COVID-19 worry can worsen the relationship between cognitive stress and psychological safety, supervisor support only directly affects psychological safety. This study contributes to expanding the stress and psychological safety literature and informs practitioners in healthcare organizations about how to deal with cognitive stress in the “*new normality*” that the pandemic has brought.

## Introduction

The COVID-19 pandemic has placed health professionals all over the world in an unprecedented situation, working under extreme pressures, both physically and psychologically (Greenberg et al., 2020; Howe et al., 2020). The new reality that the pandemic has brought has generated consequences that both organizations and individuals must face. Many people are adjusting to the new organizational demands the pandemic has caused while hoping to *“return to normal”* as soon as possible. However, the implications of these demands have provoked a much greater change, in which a more aptly named *“new normality”* has arisen. For example, the World Health Organization (WHO) has encouraged organizations to generate a work plan based on shifts and working from home, among other practices, to increase social distancing and prevent the spread of the virus (Pan American Health Organization, 2020). The changes have affected employees’ job security, financial stability, and work-family balance, especially among healthcare workers. More importantly, a less mentioned consequence has been the emotional impact that these changes have generated in employees, especially the stress they face when it comes time to go to work.

Work stress is one of the organizational responses that most impacts workers’ mental and physical health, especially in service organizations (Kim et al., 2011). González et al. (2004) found that, on comparing the stress level between health and non-health workers, the former workers suffer from more stress. In this respect, even before the COVID-19 pandemic, health care professionals were already working in high stress environments. Long hours and shift work, intensive job demands, lack of adequate resources, and fatigue are among the typical factors that make healthcare organizations environments where stress, emotional exhaustion and burnout prevail (Rice et al., 2014; Zhou and Chen, 2021). In some situations, low wages, professional invalidation, and limited career progression intensify the problem, decreasing the job satisfaction and leading workers to leave their organizations (Rice et al., 2014). Unfortunately, the COVID-19 pandemic has put these workers in an even worse situation (Greenberg et al., 2020). During the pandemic, the fear of becoming infected and dying from the virus, along with the fear of infecting family, friends, and colleagues (Monterrosa-Castro et al., 2020), has been a factor that has caused important emotional consequences in workers and increased their level of stress. The stress caused by work exhaustion and overload, often a product of long shifts and the need to cover for the absence of peers, negatively affects the quality and stability of the organization and can lead to greater dissatisfaction and intentions to leave their job (Kim et al., 2011). Stress can have significant effects on work performance, but much more importantly, on the attitudes that the workers have concerning their job stability.

Staff turnover in health care has important consequences for the organizations and the provision of quality care for patients (Gamero-Burón, 2010; Ravangard et al., 2019; Sharif et al., 2021). Hwang and Chang (2008) explain that high turnover in health care implies additional costs for human resource management and recruitment (e.g., in time and effort to employ new staff and train them for their job). Furthermore, turnover also increases the pressure on staff to work above and beyond their contracted hours, leading to errors and a decreased morale of the remaining staff (Fasbender et al., 2019). For these reasons, turnover intentions—a precursor of turnover itself (Lee and Kim, 2020)—have gained important attention throughout the years, as it could be crucial for the organizations’ productivity and the workers’ well-being.

Turnover intention refers to employees’ awareness or thoughts about leaving their job (Akgunduz and Eryilmaz, 2018). Previous research has provided evidence that a stressful working culture and increasing job demands are major contributing factors that increase employees’ intentions to leave their job (Buchbinder et al., 2001). An overburdened system, lack of support at work—especially from the supervisors, and scarce resources directly affect the emotional well-being of the employees, and certainly reduce their motivation (Flinkman et al., 2008). Therefore, the relationship between stress and turnover intentions for health care professionals needs to be revisited in the context of the current sanitary situation. By understanding this relationship, organizations can take actions to support their employees, improve their well-being, and maintain their productivity in this “*new normality*.”

To face workers’ stress and the constant changes associated with the sanitary crisis, organizations must place emphasis on the organizational processes that increase workplace stability for workers and their capacities to cope with the crisis (Lee, 2021; Oksanen et al., 2021). In this context, the individual perceptions of the workers with respect to safety in their workplace become more relevant. By feeling safe in the work environment and not exposed to inter-personal risks, workers can feel less stress and reduce the emotional and cognitive consequences it brings. In particular, psychological safety can be an important mechanism to reduce stress by creating a climate of trust and risk-free communication. Psychological safety refers to workers’ perception of how workmates can respond to the risky behaviors that interpersonal situations imply (Edmondson, 1999; Carmeli and Zisu, 2009; Frazier et al., 2017). In this way, psychological safety could translate into a mechanism that reduces workers’ stress, diminishing the negative attitudes toward their work position, that is, their intentions to leave the job.

Thus, to advance in understanding the relationship between stress and voluntary turnover, this work seeks to investigate the role of psychological safety as a mechanism that reduces the negative effects of stress on turnover intention. In particular, we focus on cognitive-type of stress, i.e., stress that generates cognitive deficits in information processing (Amirkhan et al., 2018), as this has important consequences in high-intensity jobs (Mauno et al., 2019). Cognitive stress can have significant effects on work performance, caused by the wear and tear of cognitive functions such as memory and concentration (Kalakoski et al., 2020). Therefore, our research questions are as follows: *Does psychological safety have a mediating effect between cognitive stress and workers’ turnover intention? If it does, what factors could increase or decrease psychological safety?*

Given that psychological safety could positively impact the relationship between stress and workers’ turnover intention, knowing the factors that worsen or benefit psychological safety is vital. On one hand, we evaluate the effect that worry about COVID-19 would have as a moderator between stress and psychological safety. Workers that are more worried about the consequences of COVID-19 in their lives could experience greater cognitive stress that could, in consequence, diminish their perception of psychological safety. Worry about the effects of COVID-19 has been shown to impact workers’ well-being in many aspects (Greenberg et al., 2020).

On the other hand, the organizational capacity to manage dynamics that favor a climate of psychological safety for employees could become a factor that reduces the consequence of stress in workers and allows them to feel safer when working with others (Lee, 2021). In particular, we evaluate the effect of supervisor support as a possible counter to the harmful effects of stress on perceived psychological safety. Perceiving greater support from a supervisor translates into a belief that the organization values the contributions of its employees and is concerned about their well-being (Eisenberger et al., 2002) and, therefore, could increase the perception of interpersonal safety. Previous studies on psychological safety indicate that factors such as leadership and organizational norms signal what is expected and acceptable within the organization and therefore affect how individuals perceive their work environment as safe for self-expression. In this sense, a leadership style that values the contribution of others is related to higher employee expectations that expressing oneself is acceptable in the organization (Kim, 2019). This study attempts to understand the association between cognitive-type stress and workers’ turnover intention, the possible mediating effect of perceived psychological safety and the potential moderation of worry about COVID-19 and supervisor support in the relationship between cognitive stress and psychological safety. This study aims to contribute to understanding workers’ stress in healthcare organizations, especially in the sanitary context we are facing, as well as the factors that increase/diminish those effects and their consequences in the turnover of workers. Thus, we first explain the primary relationships between the aforementioned variables to then develop hypotheses among them. Later, the hypotheses are tested on a sample of 146 workers distributed in 21 teams in different healthcare organizations. Finally, we discuss the results and implications of our findings.

## Theoretical Framework

### Psychological Safety Between Stress and Turnover Intention

Stress has been considered a complex psychological state that is produced in the interaction between the individual and the situation, in which the individual faces an imbalance between the demands of the situation and his or her capacity to respond to them (Di Martino, 1992; Cox, 1993; Gamero-Burón, 2010). These signs grow in work contexts where the rhythm of work is intensified and accelerated (Mauno et al., 2019), as is the case in healthcare organizations during the current sanitary crisis. A study done on health professionals in 34 hospitals in China reported that 50.4% of the workers had depressive symptoms, 44.6% anxiety, 34% insomnia, and 71.5% had some type of adverse reaction due to stress, where those most affected were workers who offer frontline medical attention to patients linked to COVID-19 (Wang et al., 2020). Specifically, an important manifestation of stress in these contexts is expressed in difficulties of a cognitive type (Amirkhan et al., 2018). Problems including difficulties in remembering work business, indifference toward tasks, information overload, and deterioration of the capacity to concentrate (Kalakoski et al., 2020) present themselves as manifestations of stress in these organizations.

The evidence has shown that stress does not only affect the quality of service or task performance in workers, but also can translate into negative attitudes toward the job, threatening job stability and increasing the intentions to leave the organization (Kim et al., 2011). Turnover intention is described as the conscious and deliberate freedom to leave an organization (Guimaraes, 1997). The relationship between the stress of the employees and turnover intentions has been an important focus for administrators and researchers (Qureshi et al., 2013). Different studies have attempted to identify the stress factors that have the strongest relationship with turnover intention, given that the emotional state of the workers has a considerable influence on turnover in the workplace (Wofford et al., 1999).

Today, the COVID-19 pandemic has increased the importance of understanding the effects of stress in organizations. Although it is known that greater stress increases workers’ turnover intentions, the factors that diminish these negative effects of stress are less well known, particularly how these can be effective in the current context of change that the sanitary crisis has brought. Therefore, organizations must use different management tools to avoid stress and decrease workers’ turnover intentions, such as creating a climate that encourages workers to express their difficulties and struggles, i.e., a psychologically safe climate.

Conservation of resources theory proposes a valuable insight into the resources that allow for coping with stress, such as leadership, social support, and resilience (Wright and Cropanzano, 1998). From this theoretical perspective, psychological safety becomes a positive resource that could help people to overcome anxiety and defensiveness, ultimately, relieving stress (Zhou and Chen, 2021). Psychological safety is known as the shared belief that an organization is safe in terms of assuming interpersonal risks (Edmondson, 1999). By providing a feeling of safety, employees can strive to change their behavior to meet the organizational challenges of a changing environment (Edmondson and Lei, 2014). Precisely, psychological safety promotes the idea that individuals focus on their work goals, experience fewer distractions, and solve problems more effectively, and thereby increasing their confidence to contribute significantly to organizational outcomes (Ayala Calvo and García, 2018; Zhou and Chen, 2021).

Psychological safety has been demonstrated to have positive impacts in the workplace (Frazier et al., 2017; Newman et al., 2017), including greater job stability, as well as increasing the workers’ capabilities to cope with crises (Lee, 2021; Oksanen et al., 2021). Even though it is traditionally seen as a variable at a group level, the origins of psychological safety point to workers’ individual perceptions and how safe they feel when they interact with others (see Kahn, 1990; Schein, 1993). These differences in focus have led to the construct being relevant on different levels (see Frazier et al., 2017). However, all coincide that a high level of psychological safety minimizes the perceptions of psychosocial risk in the workplace.

In healthcare organizations, psychological safety becomes more relevant as these are high-intensity environments, where interdisciplinary work is carried out daily, and mistakes can be made from exhaustion or miscommunication (Hunt et al., 2021). A lack of psychological safety can cause important negative consequences in the service to the clients and even more in the care of the patients (Edmondson, 1999). It has been demonstrated that in an environment of low psychological safety, employees do not speak up or ask for help when they need it because of fear of demonstrating weakness, damaging their reputation or threatening their job status (Ilies et al., 2010). On the contrary, a workplace with adequate levels of psychological safety would generate a greater openness for employees to express their fears and difficulties, with less concern for being rejected or of possible reprisals or consequences in their job status (Hunt et al., 2021). This opening generated by psychological safety could be crucial for workers to express the difficulties generated by stress at work. In the context of the current sanitary crisis, it has been revealed that to generate a work environment of greater well-being, it is necessary for employees to be able to communicate their concerns with tranquility and safety (Lee, 2020).

The individual perceptions of how psychologically safe the workplace will be is essential for workers to recognize and share opportunely when they need help and if they are having difficulties with current job demands (Edmondson, 1999). The way employees perceive their workplace will directly influence their capacity to function effectively, even more when they are part of a high-intensity work environment (Rantanen et al., 2021). When workers feel stressed or present difficulties produced by stress, how safe they perceive their workplace and the relationships with others in the organization can have substantial impacts on their well-being (Ilies et al., 2010; Zhao et al., 2020). For instance, when the stress is of a cognitive type and thus affects functions that are essential in performing tasks, such as concentration or their ability to use their knowledge (Sindi et al., 2017; Rantanen et al., 2021), being able to count on an environment that allows an open expression of these difficulties can be essential to increase their positive attitudes toward their job and the organization.

The importance of psychological safety in healthcare organizations (Edmondson and Lei, 2014; Zhao et al., 2020) makes it relevant to study how this can influence workers’ well-being. Although the cognitive alterations produced by stress in high-intensity work surroundings can lead workers to increase their desire to leave the organization, perceiving a psychologically safe environment could counteract those desires and thus, reduce turnover intentions. Therefore, we propose that,

*Hyp 1: Psychological Safety mediates the effect between cognitive stress and turnover intentions.*

### Worsening Psychological Safety: The Role of Worry Over COVID-19

The consequences of the COVID-19 pandemic have not only affected people medically, but have also had an important impact in workers’ mental health (Zhou and Guo, 2021). The outbreak of an infectious disease like COVID-19 has increased people’s stress levels, primarily through the anxiety generated by the uncertainty of the situation (Roy et al., 2020). This anxiety has worry as its main cognitive component (Lee, 2020), impacting directly on the well-being of health professionals (García-Iglesias et al., 2020; Perera et al., 2021). Unfortunately, in the context of change and rapid adaptation that the pandemic has brought to organizations, the workers’ mental well-being and particularly their worries have not always been a concern for the authorities (Zhou and Guo, 2021).

Different studies have demonstrated that the main concerns of health workers in contexts of the current COVID-19 pandemic are related to the fear of becoming infected and transmitting it to relatives or friends (Esteban-Carranza et al., 2021; Sahashi et al., 2021). It has been argued that this worry increases stress and imposes a significant emotional load on employees that is translated into greater difficulties in performing their jobs (Esteban-Carranza et al., 2021). The anxiety that emanates from the worry about the pandemic is harmful for the health and subjective well-being of workers (Malone and Wachholtz, 2018), affecting how people face interpersonal and professional relationships (Zhou and Guo, 2021). Dollard and Bakker (2010) argue that not managing concerns or not paying attention to the fears that workers face, means that they hide them instead of expressing them. Thus, worries about COVID-19 can become an underlying threat to people and an important source of additional stress in their lives.

The worry about the effects of COVID-19 in their lives and the increasing stress that arises from the nature of their task and the changing environment can affect how workers perceive their relationships with others and the risk of expressing their opinions or insecurities to them. Worry about COVID-19 can be understood as a cognitive and emotional process that can have consequences on workers’ mental health (Cedeño et al., 2020). Added to the stress experienced by workers, worry about COVID-19 could affect job perception and the relationships the workers have (Prieto-Callejero et al., 2020). Jun et al. (2021) suggest that the current worries about COVID-19 reduce the attention and cognitive capacity that must necessarily be used in important tasks. In the case of cognitive stress, the loss of concentration, distraction, or confusion (Jun et al., 2021) could be boosted by the anxiety of becoming infected or infecting others with the virus and the concern about the consequences of this situation. Therefore, worry about COVID-19 could be related to greater cognitive stress, i.e., lack of attention and cognitive difficulties in job performance, which leads to greater fear of expressing those concerns openly and relating freely with colleagues in the workplace. Thus, we propose that:

*Hyp 2: When employees are more worried about COVID-19, the negative effect of cognitive stress on psychological safety will be stronger.*

### Improving Psychological Safety: The Support Role of the Supervisor

During crisis contexts, as is the case of the current pandemic, leaders acquire a fundamental role in helping employees to overcome latent threats and fears (Ehlers and Clark, 2000). Even though the relationship between supervisors and employees changes all the time, the perception of the employees with regard to the support offered by supervisors in their tasks has important implications in employees performing their tasks efficiently (Shamir, 2011). Supervisor support is related to meeting goals and analyzing errors (Guchait et al., 2016) and increasing organizational commitment and job satisfaction (Gagnon and Michael, 2004; Carmeli and Zisu, 2009).

Some researchers have argued that the perceived supervisor support, in stressful contexts, brings with it a greater sensation of well-being and positive psychological results which allow workers to perform adequately (Aldamman et al., 2019). The support of the supervisor is transformed into a resource that allows stressful events to be processed from another perspective, seeing, for example, these situations as possible experiences for growth and development (Guchait et al., 2016; Singh et al., 2018). Thus, some researchers have already linked supervisor support in the current context of sanitary crisis with the control and mitigation of stress in workers, showing that it has positive effects in reducing stress (Andrades-Tobar et al., 2021). Supervisor support allows the reduction of employees’ emotional exhaustion, given that it diminishes the uncertainty they feel about COVID-19, and, in this way, they can manage their cognitive resources to reach their objectives (Vinokur and van Ryn, 1993; Charoensukmongkol and Phungsoonthorn, 2020). Therefore, supervisor support has also been linked to controlling the symptoms of cognitive stress in highly demanding jobs (Rantanen et al., 2021).

The change in working conditions in healthcare organizations produced by the pandemic has led leaders to have a fundamental role in guaranteeing that tasks and services are adequately carried out. Along these lines, leaders can directly contribute to creating positive surroundings with a sensation of psychological safety and lower levels of anxiety when it comes to explaining to the employees the nature of the job they do and the possibility that things can move away from what is expected (Edmondson and Lei, 2014). The sources of perceived support that are centered in a fair deal in the process of work and open communication with employees promote the psychological well-being workers need to face current changes in their workplace (for example, staggered shifts, telework, work overload) in the best way possible during the COVID-19 pandemic (Lee, 2021). Different studies have demonstrated that supervisor support is one of the organizational practices that encourages psychological safety, as it is when workers feel supported by their supervisor that the climate at work, satisfaction with the organizational surroundings, and commitment improve (Carmeli and Zisu, 2009; Chas and Fontela, 2013; Contreras et al., 2021; Del Estal-García and Melián-González, 2021).

Psychological safety, therefore, is formed through the interaction between the members of a team and its leader and is profoundly influenced by the support of supervision (Liu et al., 2020). By demonstrating individual consideration toward the employees and granting additional training and support in meeting goals and job responsibilities (Liaw et al., 2010; Lee, 2021), supervisors will contribute to the emotional and cognitive consequences of stress in the employees being less noticeable. In contexts where job demands and requirements have increased, feeling the supervisor’s support will reduce cognitive stress symptoms and thus improve the perception of psychological safety. Thus, we propose that:

*Hyp 3: When supervisors are perceived as supportive, the negative effect of cognitive stress on psychological safety will be weakened.*

**FIGURE 1:**
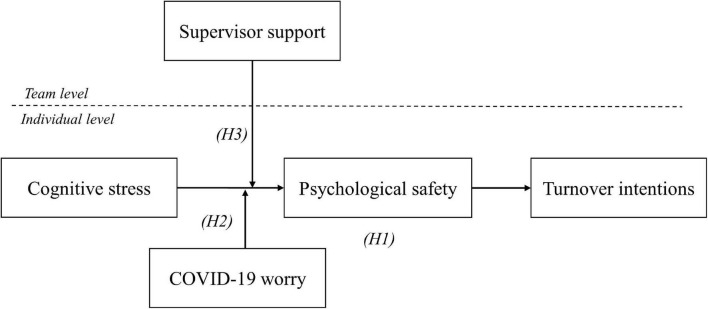
Hypothesized model.

The hypotheses are summarized in Figure 1.

## Materials and Methods

### Sample and Procedure

The hypotheses were tested using data on 146 members working in 21 teams in two public hospitals in a southern region of Chile. The hospitals are the only public medical centers in the region, so both received our invitation to participate in the study together with the corresponding documents about the procedures and the ethical guidelines. Both hospitals accepted our invitation, allowing one of the researchers to approach certain teams and invite them to join this study. Due to the protocols and restrictions given the sanitary situation of the hospitals, teams were approached by convenience, privileging administrative teams. Therefore, the sample is non-probabilistic. According to Fritz and MacKinnon (2007), for a mediation analysis with bias-corrected bootstrap and a medium to high effects, a sample of 115 participants is adequate (see Table 1, HM—Bias-corrected Bootstrap, p. 237).

**TABLE 1:** Confirmatory factor analysis hypothesized model vs. alternative model.

Factor structure model	χ^2^(*df*)	CFI	TLI	RMSEA	SRMR	Δχ^2^ (*df*)
Four factor model (hypothesized): Psychological safety, cognitive stress, turnover intentions, and supervisor support	162.68 (84)	0.93	0.92	0.08	0.06	
Three factor model (alternative): Psychological safety and supervisor support constrained as one factor	222.364 (87)	0.89	0.86	0.1	0.09	59.68 (3)
Two factor model (alternative): Psychological safety and supervisor support constrained as one factor and cognitive stress and turnover intentions constrained as one factor	372.3 (105)	0.76	0.72	0.15	0.13	209.62 (5)

*N = 145. All χ^2^ and Δχ^2^ values are significant at p < 0.05. CFI, comparative fit index; TLI, Tucker-Lewis index; SRMR, standardized root-mean-square residual; RMSEA, root-mean-square error of approximation.*

Participants work full time on administrative and healthcare-related tasks. Given the nature of their tasks, the teams require intense coordination and communication of their members with each other. Employees were invited to participate in this study without being offered any type of compensation. Before completing the survey, the research team required the participants’ consent and informed them that the study and the consent had been approved by the research ethics committee at the University where one of the authors of this paper is employed.

In total, surveys were distributed to 156 employees from 21 teams. However, due to missing data, 10 respondents were excluded. While 89% of the participants work on administrative duties, 11% of the participants work in healthcare functions. Teams range from 3 to 13 members, with an average of 7.43 members (*SD* = 3.44). Among the respondents, 82 were female (53%). In terms of age, 30% of the participants were between 18 and 30 years of age, 43% were between 31 and 40 years of age, 18% were between 41 and 50 years of age, and the remaining 9% were above 50 years of age. Regarding tenure, 20% of the participants have been in their organization less than 1 year, 22% between 1 and 3 years, 13% between 3 and 5 years, and the remaining 46% above 5 years.

### Measures

Cognitive stress was measured with a 3-item scale of cognitive stress developed by Madrid (2020). Participants were asked to what extent they (1) “*have problems concentrating*”; (2) “*have difficulties remembering things*”; and (3) “*have trouble keeping their attention on their tasks*.” Answers range from 1 (*never*) to 5 (*always*). The Cronbach’s alpha for this scale was 0.86.

Psychological safety was measured with a 5-item scale adapted from Edmondson’s (1999) psychological safety scale. Items include “*If you make a mistake in this team, it is often held against you*,” and “*It is safe to take risks on this team*.” Answers ranged from 1 (*strongly agree*) to 5 (*strongly disagree*). The Cronbach’s alpha for this scale was 0.71. We decide not to aggregate the measure of psychological safety at the team level, as our aim was to capture the individuals’ perception of safety and ability to manage change while working with members of their teams. As Newman et al.’s (2017) meta-analysis confirmed, an important number of studies have used individually held rather than aggregate perceptions to capture psychological safety.

Supervisor support was measured with the 4-item scale from Haynes et al. (1999). Participants were asked ranging from 1 (*to a very little extent*) to 5 (*to a great extent*), how much their immediate supervisors (1) “*encourage you to give your best effort?*” (2) “*help you with a difficult task at work?*” (3) “*offer new ideas for solving job-related problems?*” and (4) “*encourage those who work for him/her to work as a team?*” The Cronbach’s alpha for this scale was 0.94.

To compose a team level measure of Supervisor Support we checked the within-group homogeneity and between-group heterogeneity. We calculated the rwg_(j)_ to assess within-group homogeneity (James et al., 1993), the intraclass correlation coefficient (ICC[1]) to understand the proportion of the variance that is explained by team membership, and the ICC[2] to assess the reliability of the team means for the study variables (Bliese, 2000; Shieh, 2016). The scores for Supervisor Support are above accepted cut-off values (George, 1990), rwg_(j)_ = 0.83 (SD = 0.22), ICC[1] = 0.39, and ICC[2] = 0.83. Therefore, we proceeded to aggregate the scores to create a team level measure of Supervisor support.

COVID-19 worry was measured using a single item validated by the United Kingdom Office of National Statistics population surveys (Office for National Statistics [ONS], 2020): “*How worried or unworried are you about the effect that COVID-19-19 is having on your life right now?”* Answers ranged from 1 (*Not at all worried*) to 5 (*Very Worried*). As Fisher et al. (2016) argued, single-item measures can offer important advantages to capture conflicting constructs while minimizing non-response bias.

Turnover intentions were measured with the 3-item scale developed by Colarelli (1984). Example items include “*I frequently think of quitting my job*” and “*I will be working for this organization one year from now*” (reverse scored). Answers ranged from 1 (*strongly agree*) to 5 (*strongly disagree*). The Cronbach’s alpha for this scale was 0.77. Measurements of turnover intentions were non-independent in teams, ICC[1] = 0.07, *F*_(20,__135)_ = 1.51, *p* < 0.10. Teams were also somewhat distinguishable by their average level of turnover intentions, ICC[2] = 0.34.

### Control Variables

We also accounted for three different types of individual characteristics which might impact psychological safety and turnover intentions: gender (1 = *female*, 0 = *male*), age, and tenure. Since we could expect differences in turnover intentions depending on the responsibilities of the employees, that is, whether they have other people in charge, we also controlled by whether participants have or not a managerial role (1 = *manager role*, 0 = *no manager role*). We finally control for the nature of the task of the participants (1 = *healthcare tasks*, 0 = *administrative tasks*).

### Analytic Strategies

We calculate descriptive statistics and correlations using the psych package in R (Revelle, 2021). Before testing the hypotheses, we perform a Confirmatory Factor Analysis (CFA). This analysis allows to check the fit of the observed data to the proposed scales mentioned in the measures section (Mueller and Hancock, 2001).

Furthermore, due to the hierarchical organization of the data, we tested the hypotheses with mixed models using the lme4 package (Bates et al., 2014) of the R environment. The analysis spanned two levels: the individual level and the team level. We fitted simple models and added random effects to identify the best fitting model by comparing model fit indices (i.e., the Akaike information criterion (AIC) and Bayesian information criterion (BIC) criteria). The random intercept-and-slope model did not fit the data better than the random-intercept-only model, Δχ^2^(2) = 0.03, *p* = 0.98. Hence, we employed a random-intercept-only model for further hypothesis testing.

In addition, we used the package mediation (Tingley et al., 2014) to test the indirect effect of psychological safety on the relationship between stress and turnover intentions as proposed in Hypothesis 1.

#### Confirmatory Factor Analysis

Confirmatory factor analyses^1^ were conducted to examine whether employees’ scores on their self-report measures (i.e., psychological safety, cognitive stress, supervisor support, and turnover intentions) captured distinctive constructs. The hypothesized four-factor model was specified by loading indicators on their respective latent variables, and the correlations among latent variables were freely estimated. The results showed that the four-factor model fits the data well, χ^2^(84, *N* = 145) = 162.68, comparative fit index (CFI) = 0.93, Tucker—Lewis index (TLI) = 0.92, standardized root-mean-square residual (SRMR) = 0.06, and root-mean-square error of approximation (RMSEA) = 0.08. The indicators all significantly loaded on their respective latent factors. As an additional test, we compared the hypothesized four-factor model with several alternative models, as shown in Table 1. All the alternative models fit the data significantly worse than the four-factor model. Therefore, we can conclude that the measures reported by employees captured distinct constructs in this study.

## Results

The means, standard deviations, and bivariate correlations among the studied variables are shown in Table 2. At the individual level, cognitive stress is positively correlated with employees’ turnover intentions (*r* = 0.22, *p* < 0.05), while psychological safety is negatively correlated with individuals’ turnover intentions (*r* = −0.36, *p* < 0.01) and cognitive stress (*r* = −0.27, *p* < 0.01). The magnitude of the correlation coefficients suggested that these relationships were generally moderate to medium (Hemphill, 2003). These results mirrored findings from previous research that found moderate correlations between psychological safety and turnover intentions, and stress and turnover intentions (Mosadeghrad, 2013; Kruzich et al., 2014; Kirk-Brown and van Dijk, 2016). Before further analysis, we mean-centered the independent variables.

**TABLE 2:** Descriptive statistics and correlations.

	Average	SD	1	2	3	4	5	6	7	8	9	10
**Level 1—Individual level**												
1. Gender (1 = Female)	0.54	0.50										
2. Age	2.05	0.90	0.14									
3. Tenure	2.86	1.20	0.05	0.48								
4. Manager role (1 = Yes)	0.22	0.42	–0.01	0.21*	0.26**							
5. Healthcare role (1 = Yes)	0.12	0.32	0.03	0.10	–0.01	0.32**						
6. Turnover intentions	2.11	0.98	–0.12	–0.11	–0.01	–0.03	0.00	(0.77)				
7. Cognitive stress	2.31	0.86	0.08	0.03	0.11	–0.02	0.17*	0.22*	(0.86)			
8. Psychological safety	3.91	0.79	0.04	–0.07	−0.17*	0.04	0.00	−0.36**	−0.27**	(0.71)		
9. COVID-19 worry	4.13	0.86	–0.02	0.13	0.05	–0.08	0.12*	0.05	0.08	0.01	__	
**Level 2—Team level**												
10. Supervisor support	3.90	0.79										(0.94)

N level 1 = 146. N level 2 = 21. *p < 0.05; **p < 0.01. Internal consistency coefficients, Cronbach’s alphas are reported in the parentheses on the diagonal.

### Hypothesis Testing

Hypothesis 1 predicted that psychological safety will mediate the effect of stress on turnover intentions. First, results in Model 1 (Table 3) show that stress has a positive and significant direct effect on turnover intentions (*b* = 0.23; SE = 0.09, *p* < 0.05, 95% BTCI = [0.03, 0.41]). From Model 2 in Table 3, we found that stress has a negative and significant effect on psychological safety (*b* = −0.20; SE = 0.07, *p* < 0.01, 95% BTCI = [−0.35, −0.05]). Model 3 in Table 3 shows that when together, stress has a positive yet not significant effect on turnover intentions (*b* = 0.17; SE = 0.09, *p* > 0.05, 95% BTCI = [−0.01, 0.34]), while psychological safety has a negative and significant effect on turnover intentions (*b* = −0.36; SE = 0.10, *p* < 0.001, 95% BTCI = [−0.58, −0.17]). To confirm a mediation effect, we followed Hayes (2018a,b) suggestions and tested a potential indirect effect through bootstrapping estimation of a confidence interval. If the confidence interval does not include zero, then we could confirm the existence of a mediation. Our results support an indirect mediation effect of stress on turnover intentions via psychological safety (b = 0.07; 95% BTCI = [0.01, 0.15]), as the bootstrapped confidence interval does not include zero. We found that roughly 30% of the effect from stress on turnover intentions goes through psychological safety. Hence, these results support Hypothesis 1.

**TABLE 3:** Multilevel mediation analysis random-intercept-only model.

	Model 1				Model 2				Model 3			
	DV: Turnover intentions				DV: Psychological safety				DV: Turnover intentions			
	Est.	SE	95% BTCI		Est.	SE	95% BTCI		Est.	SE	95% BTCI	
1. Intercept	2.25***	(0.14)	1.95	2.56	–0.12	(0.12)	−0.36	0.14	2.20***	(0.13)	1.97	2.45
2. Age	–0.13	(0.10)	−0.32	0.05	–0.003	(0.08)	−0.16	0.16	–0.14	(0.10)	−0.34	0.04
3. Tenure	0.04	(0.08)	−0.10	0.20	–0.1	(0.06)	−0.22	0.04	0.01	(0.08)	−0.15	0.17
4. Gender (1 = Female)	–0.25	(0.16)	−0.53	0.03	0.2	(0.13)	−0.04	0.45	–0.18	(0.15)	−0.48	0.08
5. Manager role (1 = Yes)	–0.05	(0.21)	−0.49	0.37	0.17	(0.17)	−0.17	0.48	0.02	(0.20)	−0.43	0.45
6. Healthcare role (1 = Yes)	–0.003	(0.32)	−0.63	0.64	–0.08	(0.28)	−0.64	0.42	–0.03	(0.30)	−0.61	0.50
7. Cognitive stress	0.23*	(0.09)	0.03	0.41	−0.20**	(0.07)	−0.35	–0.05	0.17	(0.09)	−0.01	0.34
8. Psychological safety									−0.36***	(0.10)	−0.58	−0.17
AIC	430.49				366.43				423.65			
BIC	457.46				393.40				453.62			
Pseudo-R-squared*^a^*	0.07				0.08				0.15			
Log likelihood	–206.24				–174.21				–201.82			
Num. obs.	146				146				146			
Num. groups	21				21				21			
Var: Team (Intercept)	0.08				0.01				0.03			
Var: Residual	0.84				0.51				0.80			

		**95% BTCI**										
	**Est.**	**Lower**	**Upper**									

Indirect effect	0.07	0.01	0.15									
Direct effect	0.17	−0.02	0.35									
Total effect	0.24	0.05	0.42									
Proportion mediated	0.30											

***p < 0.001; **p < 0.01; *p < 0.05. BTCI, 95% Bootstrap Confidence Interval using 10,000 samples. ^a^We estimated the overall variance explanation of the random-intercept-only models with the pseudo-R-squared for generalized mixed-effect models (Nakagawa et al., 2017).

**FIGURE 2:**
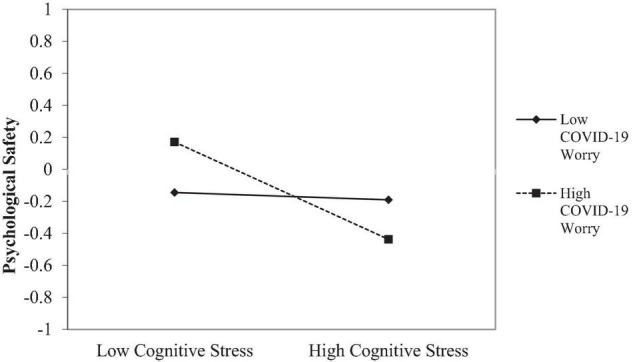
Moderation of COVID-19 worry on the effect of cognitive stress on psychological safety.

Hypotheses 2 and 3 propose a moderation effect of worry about COVID-19 and supervisor support, respectively, on the effect of stress on psychological safety. Model 2 in Table 4 shows the results for the moderating effect of COVID-19 worry on the relationship between stress and psychological safety (Hypothesis 2). The interaction of stress and COVID-19 worry has a negative and significant effect on psychological safety (*b* = −0.19; SE = 0.09, *p* < 0.05, 95% BTCI = [−0.38, −0.01]), after controlling for supervisor support. The interaction of COVID-19 on the effect of stress on psychological safety is illustrated in Figure 2. Higher levels of stress combined with a deep worry for COVID-19 will have a more negative effect on the team members psychological safety.

**TABLE 4:** Multilevel moderation analysis random-intercept-only model.

	Model 1				Model 2				Model 3			
	DV: Psychological safety				DV: Psychological safety				DV: Psychological safety			
	Est.	SE	95% BTCI		Est.	SE	95% BTCI		Est.	SE	95% BTCI	
**Level 1—Individual**												
1. Intercept	–0.14	(0.11)	−0.34	0.06	–0.16	(0.11)	−0.37	0.05	–0.13	(0.11)	−0.33	0.07
2. Age	0.01	(0.08)	−0.14	0.17	0.03	(0.08)	−0.12	0.19	0.01	(0.08)	−0.14	0.17
3. Tenure	–0.08	(0.06)	−0.21	0.04	–0.08	(0.06)	−0.20	0.04	–0.08	(0.06)	−0.20	0.05
4. Gender (1 = Female)	0.21	(0.12)	−0.05	0.45	0.25*	(0.13)	0.00	0.49	0.21	(0.13)	−0.04	0.45
5. Manager role (1 = Yes)	0.16	(0.16)	−0.15	0.48	0.21	(0.16)	−0.11	0.53	0.16	(0.17)	−0.13	0.49
6. Healthcare role (1 = Yes)	0.11	(0.23)	−0.35	0.58	0.08	(0.23)	−0.38	0.54	0.11	(0.23)	−0.34	0.53
7. COVID-19 worry	0.03	(0.07)	−0.13	0.16	0.02	(0.07)	−0.12	0.16	0.03	(0.07)	−0.11	0.18
8. Cognitive stress	−0.18*	(0.08)	−0.31	–0.03	–0.14	(0.08)	−0.29	0.00	−0.17*	(0.08)	−0.32	−0.02
9. Cognitive stress * COVID-19 worry					−0.19*	(0.09)	−0.38	−0.01				
**Level 2—Team**												
10. Supervisor support	0.35***	(0.10)	0.16	0.53	0.36***	(0.10)	0.17	0.55	0.34***	(0.10)	0.16	0.55
**Cross-level interaction**												
11. Cognitive stress * Supervisor support									0.05	(0.11)	−0.17	0.28
AIC	360.83				3361.65				365.23			
BIC	393.65				397.45				401.03			
Pseudo-R-squared*^a^*	0.20				0.23				0.20			
Log likelihood	–169.42				–168.82				–170.62			
Num. obs.	146				146				146			
Num. groups: TEAM	21				21				21			
Var: Team (Intercept)	0.024				0.027				0.026			
Var: Residual	0.510				0.497				0.512			

***p < 0.001; *p < 0.05. BTCI, 95% Bootstrap Confidence Interval using 10,000 samples. ^a^We estimated the overall variance explanation of the random-intercept-only models with the pseudo-R-squared for generalized mixed-effect models (Nakagawa et al., 2017).

Regarding Hypothesis 3, Model 3 in Table 4 shows the results for the moderating effect of supervisor support on the relationship between stress and psychological safety. Supervisor support does not moderate the effect of stress on psychological safety (*b* = 0.05; SE = 0.11, *p* > 0.05, 95% BTCI = [−0.17, 0.28]), yet it has a positive and significant direct effect on psychological safety (*b* = 0.34; SE = 0.10, *p* < 0.001, 95% BTCI = [0.16, 0.55]), after controlling for stress.

Therefore, although our results support Hypothesis 2, we could not confirm Hypothesis 3. Table 5 summarizes the tested and confirmed hypotheses.

**TABLE 5:** Summary of hypotheses and results.

Hypotheses		Results
1	*Psychological Safety mediates the effect between cognitive stress and turnover intentions.*	Confirmed
2	*When employees are more worried about COVID-19, the negative effect of cognitive stress on psychological safety will be stronger.*	Confirmed
3	*When supervisors are perceived as supportive, the negative effect of cognitive stress on psychological safety will be weakened.*	Not confirmed

## Discussion

This study aimed to understand the effect of cognitive stress on turnover intentions as well as the mediating effect of perceived psychological safety in this relationship, in addition to determining whether supervisor support and COVID-19 worry have a moderating role in the effect of cognitive stress on psychological safety.

The results of the multilevel mixed models show that perceived psychological safety mediates the relationship between cognitive stress and turnover intentions. Furthermore, we found that COVID-19 worry moderates the relationship between cognitive stress and perceived psychological safety, such that higher COVID-19 worry worsened the relationship between cognitive stress and psychological safety. Although not expected, we did not find evidence of the moderation of supervisor support on the relationship between cognitive stress and perceived psychological safety. However, we did find a direct and positive effect of supervisor support on the psychological safety perceptions.

The results of the mediating analysis confirm that higher perceived psychological safety prevents the negative effects of cognitive stress on turnover intentions, thus confirming our first hypothesis. Therefore, we contribute to the literature on stress as we explore a potential mechanism that can prevent its negative effects on employees’ attitudes toward their job. These results are consistent with previous research (Edmondson, 1999; Frazier et al., 2017; Lee, 2021), which states that an individual’s perceptions of psychological safety reduce the potential risk associated with interacting with others at work and, in particular, the potential adverse effects that stress could have when working with others. Considering psychological safety as a mechanism that prevents the negative consequences of stress at work offers important opportunities to deal with cognitive stress, especially regarding the immediate effects that this has, such as loss of concentration, distraction, or confusion (Jun et al., 2021). In situations that create or reinforce cognitive stress for employees, processing information and performing tasks will be more challenging (Amirkhan et al., 2018; Kalakoski et al., 2020); hence creating an environment that allows employees to express their difficulties and challenges when performing their tasks increases the opportunities to take action and allows employees to perform their jobs accordingly. Psychological safety can become an important mechanism to manage and counteract the stressful consequences of the current sanitary crisis and the constant change the “*new normality*” (Oksanen et al., 2021) has brought to organizations.

The relationship between stress and turnover intention is not always straightforward because it depends on the employees’ perceptions of threats (Sindi et al., 2017). Psychological safety can explain why employees’ stress changes according to the context in which they perform their jobs, such that on many occasions being in an environment that allows them to express themselves freely and without fear of being embarrassed or punished can reduce their negative attitudes toward their job (Frazier et al., 2017).

Our results also contribute to a better understanding of how stress, particularly cognitive type stress, affects turnover intentions. Most studies on stress and its effects on turnover intentions focus on a chronic type of stress, particularly burnout (Hayes et al., 2012; Chênevert et al., 2021); and fewer have addressed the relationship with cognitive symptoms of stress (e.g., working memory difficulties, indifference to work, information overload and impaired concentration) (Rantanen et al., 2021). Cognitive stress has significant effects on the quality of attention and the performance of workers at healthcare organizations, as it increases the likelihood of errors at work and impairs the sense of self-efficacy, which has also been linked to increased intentions to quit (Buchbinder et al., 2001).

Our study also tested the potential moderating effects of COVID-19 worry and supervisor support on the relationship between cognitive stress and psychological safety. While we found that COVID-19 worry worsens the adverse effects of cognitive stress on employees’ perceptions of psychological safety, we could not prove that supervisor support had a moderating effect on this relationship.

In situations that generate high stress, as is the case of the COVID-19 pandemic, the levels of anxiety that people experience are translated into a great concern about the potential negative effects that these could for them and their surroundings. Such a situation creates a context where stress (of any type) is more likely to increase. As Jun et al. (2021) argued, stress creates an automatic sense of anxiety and concern, affecting the cognitive control required for performing tasks and functions. The fear and the insecurities that emerge in situations characterized by high distress (as it is the case of the pandemic for healthcare workers) reinforce those threats that employees perceive of being rejected or judged by their peers because they cannot perform their task as they would be in a normal situation (Ilies et al., 2010). Therefore, the employees’ perceptions of psychological safety are affected, creating an environment where workers cannot give the best. Furthermore, these negative consequences could affect the employees’ attitudes toward their job, increasing their turnover intentions in the long-term.

Although we could not confirm that supervisor support moderated the relationship between cognitive stress and perceived psychological safety, we found that supervisor support has an important contribution in creating a psychologically safe environment. That is, our results show a direct and positive effect of supervisor support on perceived psychological safety. The literature on supervisor support for stress management is nascent (Horan et al., 2018). Most of the studies have attempted to show that low levels of supervisor support contribute to increased stress and even turnover, paying less attention to the moderating effects on these variables and other organizational processes (Bélanger et al., 2015; Meral et al., 2018). In this respect, the lack of the moderation effect can be explained in two ways. First, for supervisor support to be effective in reducing employees’ stress, supervisors should be concerned about their workers’ stress levels specifically and make stress management a priority (Kath et al., 2012). Effective stress management will only be possible when direct actions are taken in order to alleviate the factors that cause stress in the employees and the work environment. Precisely, Lee (2021) states that supervisor support becomes an organizational resource only once it means that employees feel listened to and considered. Alternatively, for cognitive type stress, a more task-focused supervisor support could diminish the employees’ concerns regarding their job. Higher orientation, attention, and feedback from the supervisor to workers in relation to their work tasks can be more effective rather than a general support (Eisenberger et al., 2002).

Previous studies have also confirmed the association between supervisor support and psychological safety (Edmondson and Lei, 2014). Supervisors can enhance open communication and facilitate employees to express their concerns (Singh et al., 2018). Systematic support from supervisors to employees enhances the knowledge of the current conditions that the employees are undergoing at work, whilst at the same time reducing the uncertainty that a crisis or constant change could create (Singh et al., 2018). Although we could not prove that supervisor support can be necessary for reducing the negative effects of cognitive stress on psychological safety, increasing the support that supervisors give to employees in the context of the COVID-19 pandemic can certainly make them feel safe to express their concerns and difficulties.

Overall, our findings provide evidence of cognitive stress’s consequences for employees working at healthcare organizations in situations of high distress, as it is the case of the current pandemic. Although not as evident as other types of stress, cognitive difficulties that arise from the anxiety that employees experience at work can seriously undermine their relationships with colleagues and, most importantly, their attitude toward their job. Therefore, we contribute to the stress literature by showing concrete evidence of how cognitive stress affects turnover intentions in healthcare organizations. Furthermore, our findings also contribute to the psychological safety literature by providing direct evidence of how cognitive stress affects employees’ perceptions of psychological safety and, at the same time, how psychological safety can counteract the negative effects of stress on turnover intentions. Psychological safety is a crucial variable for increasing learning, engagement, and performance among workers (Frazier et al., 2017), especially in the constant change that organizations experience. We also tested two variables that have a contingent effect on the relationship between stress and psychological safety, showing how COVID-19 worry combines with stress to undermine psychological safety perceptions. Finally, we tested our hypotheses in a sample collected during the crisis that COVID-19 has brought to the healthcare organizations. Therefore, we capture real-time perceptions and emotions of the employees in these organizations.

## Implications and Conclusion

The study results point out that the employees’ cognitive stress has a significant impact on their intentions to leave the organization. Health care workers are consistently exposed to factors that produce stress (e.g., overload, lack of adequate resources, exhaustion, etc.), and it is undeniable that the COVID-19 pandemic has increased the sources of stress. The constant threat of the consequences of the virus and the emotional exhaustion that carries have direct effects on the employees’ capacity to use their knowledge, perform their tasks according to expectations and collaborate with others. Therefore, in these times of change, healthcare organizations should look deeper at the consequences of stress and the mental health of their employees and the processes that reduce their impact on the employees’ work and well-being, e.g., psychological safety.

As the results of this study show, psychological safety can play an important role in preventing the negative consequences of cognitive stress on turnover intentions. Prior to the pandemic, Nembhard and Edmondson (2006) found that within health care teams, psychological safety is a key factor to promote workers speaking up and learning behaviors. Practitioners in the field of people management in healthcare organizations should bear in mind that psychological safety could be an important factor in creating a safe and protected environment for the employees to express their concerns. More importantly, in this “new normality,” psychological safety can be a catalyst of important organizational processes, by contributing to reducing employees’ dissatisfaction with their job (as reflected in less intentions to leave their organization).

Managers should place emphasis on the practices that increase psychological safety. First, reducing the threat that COVID-19 presents for employees can have important effects in reducing the negative effects of cognitive stress on psychological safety as our study shows. For instance, Khan (2021) shows that social media disinformation has a positive relationship with the threat of COVID-19 in healthcare workers. By introducing reliable sources and clear communication, managers can help to reduce employees worries about the pandemic. Further, promoting social distance, and utilizing alternative channels for work and communication (e.g., webinars, social media platforms, and video calls) can also contribute to the feelings of safety for the employees. The threat that COVID-19 presents for health care workers should continue to be studied as its effects on different spheres are still uncertain. Future research can include other individual differences as moderators of the worry for the COVID-19, such as personality and locus of control.

Second, supervisor support can directly increase psychological safety. By training supervisors to give better support to employees and creating a climate with open communication and receptiveness of employees’ concerns, health care organizations can promote a climate of psychological safety. It is also essential that the supervisor support is persistent and inclusive (Nembhard and Edmondson, 2006). Supervisors should accompany the workers throughout their difficulties, encourage open communication and reduce the barriers that make employees feel excluded. Future research could look more in detail at the role of specific types of supervision in the relationship between stress and psychological safety, especially in emotionally challenging situations. When interpreting and generalizing the study’s results, caution should be kept in mind in light of the following limitations. First, we applied a single questionnaire to collect the employees’ perceptions of the main variables. This can give way to certain biases of desirability and self-report. Future studies should consider longitudinal samples, where the temporal aspects of the variables under study are considered. Second, this study was conducted in only two healthcare organizations in Chile which we accessed by convenience; this may affect the generalizability of the findings to other countries and the general population. However, our results still pose significant contributions for healthcare organizations’ managers and how they can deal with employees’ stress in the current sanitary situation and the “new normality.” Third, for this study we measured supervisor support in a general way and given our findings, future studies should consider the specific type of support given by the supervisor. Other variables related to the supervisor’s well-being and leader characteristics can also help to understand how they can contribute to reducing employees’ stress and promote psychological safety at work. Finally, although this study was implemented during the COVID-19 sanitary crisis, our results can also offer important guidelines for the management of public health organizations outside the context of the pandemic. Healthcare organizations are emotionally charged environments, where stress is inherent to the work that employees perform on a day-to-day basis.

To sum up, in this study, we aimed to understand the effects of cognitive stress on turnover intentions, showing that psychological safety can indeed prevent the adverse effects of stress on turnover intentions. We further found that COVID-19 worry increases the harmful effects of cognitive stress on psychological safety. Therefore, we found that psychological safety needs to be considered within healthcare organizations, especially during the pandemic, as this can help to reduce part of the stress that employees at these organizations experience. We trust that the knowledge elaborated here will be informative for practitioners in these organizations to look after the well-being of the employees during these times of change.

## Data Availability Statement

The original contributions presented in the study are included in the article/supplementary material, further inquiries can be directed to the corresponding author/s.

## Ethics Statement

The studies involving human participants were reviewed and approved by the Comité de ética de la Universidad Católica de la Santísima Concepción (Número 19-2020). The patients/participants provided their written informed consent to participate in this study.

## Author Contributions

MH and FT-M contributed equally to the conception, design of the study, data collection, analysis, and writing of the manuscript. KO collected part of the data and contributed to the writing of the manuscript. All authors reviewed, read, and approved the submitted version.

## Conflict of Interest

The authors declare that the research was conducted in the absence of any commercial or financial relationships that could be construed as a potential conflict of interest.

## Publisher’s Note

All claims expressed in this article are solely those of the authors and do not necessarily represent those of their affiliated organizations, or those of the publisher, the editors and the reviewers. Any product that may be evaluated in this article, or claim that may be made by its manufacturer, is not guaranteed or endorsed by the publisher.

## Footnotes

1 To perform the confirmatory factor analysis, we used Lavaan package of the R environment (Rosseel, 2012).
